# Supplementary material for: Genetically regulated gene expression and proteins revealed discordant effects
Source: PLoS One. 2022 May 23;17(5):e0268815. doi: 10.1371/journal.pone.0268815 (PMC9126407; doi:10.1371/journal.pone.0268815)
Supplement: S1 File — (PDF) [file pone.0268815.s001.pdf]

Supplemental Data

Pott et al. LWAS Olink biomarker

Supplemental Table – Legends

Supplemental Table S1: Overview of all 92 analyzed biomarker and the main results. Besides the sex-interaction, all results are reported for the combined setting.

Supplemental Table S2: Results of the hierarchical FDR over all 92 analyzed biomarkers in three setting (combined, male-specific, female-specific). The Simes P-value is the minimal FDR corrected p-value per gene (first level). FDR l2 is the second level FDR over all Simes p-values, and proteins are considered significantly associated if FDR l2 < 0.05 (hier. FDR == T). This resulted in k = 64, 54 and 48 proteins with significant associations in the combined setting, males, and females, respectively. We then used α=0.05*k/92 as significance level on the FDR corrected p-value per SNP of the first level, and report here how many SNPs are considered associated per gene. Columns regarding the all, males or females are indicated by _a, _m, or _f, respectively. NSig gives the number of associated SNPs with the protein, while NSig_indep indicates how many of them were independent using LD r^2<0.1 using LIFE-Adult as reference panel.

Supplemental Table S3: Genetic association results, after hierarchical FDR and priority pruning (n=760 unique SNPs). All other summary statistics can be downloaded at Zenodo [DOI: 10.5281/zenodo.6045694]. Due to overlapping cis-loci and priority pruning, not all lead pQTLs are listed (see Table S4 & S5 for all lead pQTLs per biomarker). As significance level α=0.05*64/92 was used on the FDR values.
EA = effect allele;
OA = other allele;
EAF = effect allele frequency;
info = imputation info score
#SNPs_tagged = number of significant SNPs that are in LD (r^2>0.1) with the respective SNP GWASCatalog = traits from the GWAS catalog including LD to catalog SNP (r^2>0.3)

Supplemental Table S4: Sex-Interaction results of significant lead pQTLs in the combined, male only and female only setting. Per SNP and best setting, the PC-adjusted statistics are reported as well (adjustment for sex, age and the first 10 genetic PCs).

Supplemental Table S5: Annotation of 64 significant lead pQTLs of the combined setting with their respective effect on gene expression (GE) in all available tissues. EQTL statistics were retrieved from GTEx v8 and LIFE, if available. We compared the effect direction of SNP on PE and GE only for significant associations on GE (p<0.05). In addition, we checked for the best associated eQTL in each tissue and estimated the linkage disequilibrium (LD) between the pair if available in 1000Genomes reference.

Supplemental Table S6: Overview of 64 proteins and the tissue specific results of the combined setting. For more information, see Tables S5, S7, S8, S9 and S10

Supplemental Table S7: Results of the co-localization analyses for all significant biomarkers and all GTEx tissues. Posterior probabilities (PP) are marked in red if PP>0.75. Not all tissues were available for all genes.
H0 = no significant association at this locus (neither biomarker nor gene expression (GE))
H1 = only significant biomarker association at this locus
H2 = only significant GE association at this locus

H3 = significant biomarker and GE association at this locus, but independent signals
H4 = significant biomarker and GE association at this locus and shared signal (co-localization)

Supplemental Table S8: Results of the MetaXcan analyses for all significant biomarkers and all GTEx v8 tissues. Z-score, effect size, and p-value are MetaXcan association results for the respective gene. Hierarchical FDR accounts for multiple testing of all genes and all tissues. Significance level is calculated on the level of the gene.
var_g: variance of GE;
r2: r^2 of the tissue models's correlation to gene's measured transcriptome (prediction performance);
pred_perf_pval: p-value of prediction performance;
n_snps ... : number of SNPs that were used in MetaXcan analysis, in the covariance matrix, and in the model, respectively.

Supplemental Table S9: Top-MetaXcan results compared to correlation in LIFE. Top-associated tissues from MetaXcan analysis with effect size and hierarchical FDR level are listed for all 64 significant biomarker from the combined setting. For 29 biomarker, no GE data was available in LIFE due to quality filtering. We used Pearson’s correlation and adjusted for sex, age, lymphocytes and monocytes ratio in the partial correlation analyses.

Supplemental Table S10: Results from the Mendelian Randomization analyses. We used the best associated eQTL per tissue from GTEx. To obtain strong instruments, we restricted the analyses on eQTLs with p-value < $5x10^{-8}$. Statistics for biomarkers were obtained from our data. The causal estimate and standard error were calculated via the ratio method and as the first two terms from the delta method, respectively. We used hierarchical FDR to adjust for multiple testing of tissue and protein.

Supplemental Table S11: Results from the Mendelian Randomization analyses on CAD. We used all lead pQTLs as instruments if they reached genome-wide signigicance ($p<5x10^{-8}$). Statistics for CAD was obtained from van der Haarst & Verweij [doi: 10.1161/CIRCRESAHA.117.312086]. The causal estimate and standard error were calculated via the ratio method and as the first two terms from the delta method, respectively. We used Bonferroni method to adjust for multiple testing.

Supplemental Table S12: Results from the MR network analyses. We used the best associated eQTL per tissue from GTEx v8 for GE on PE and GE on CAD. In addition, the results for P on CAD are displayed, using the best pQTL (see Table S11). We restricted the analyses on proteins with causal effect on CAD and which are causally affected by their tissue-specific GE. The causal estimate and standard error were calculated via the ratio method and as the first two terms from the delta method, respectively. The indirect effect is the product of GE on PE and P on CAD using different instruments in each step. The direct effect is the difference between total and indirect effect of GE on CAD.

Supplemental Figures

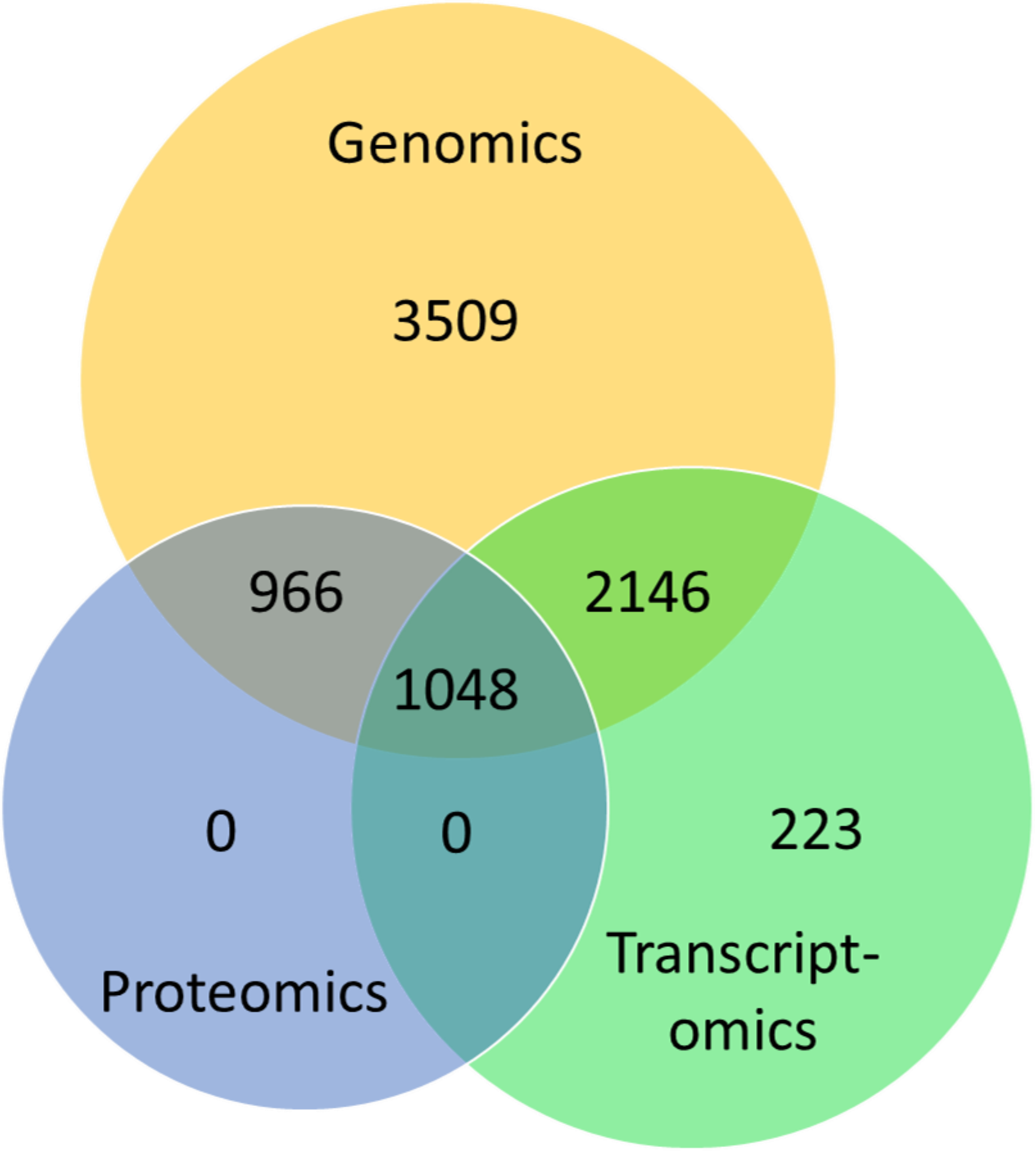

Figure S1: Sample Overview of our LIFE-Adult study. All of our samples with proteome measurements also had genetic data, i.e. a sample size of N=2,014 was available for genetic association analysis of proteome features. For the eQTL lookup in LIFE-Adult, we had a sample size of 3,194. For the GE-protein correlation look-up, a sample size of 1,048 was available.

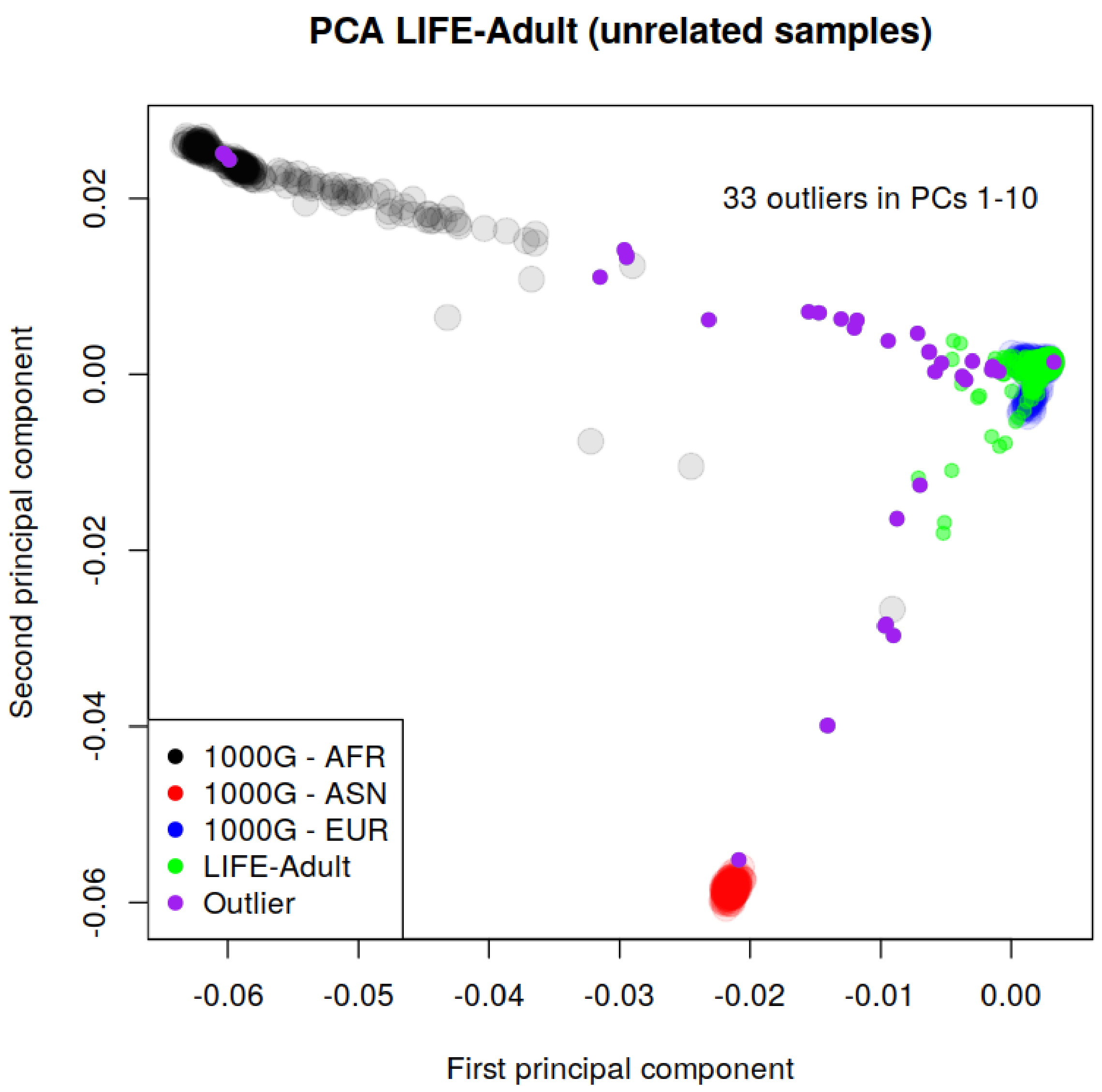

Figure S2: Principal components analysis of LIFE-Adult. The genotypes of 133,772 pruned SNPs (pairwise r^2<0.2) were used for PCA. Samples from 1000Genomes (Phase 3) and LIFE-Adult were analyzed. 1000Genomes is colored by ethnicity (black: Africans, red: Asians, blue: Europeans). Samples of LIFE-Adult are colored green, and lie within the European cluster. Outliers (>6 SD of the first ten PCs) are marked purple. Those were filtered for analysis.

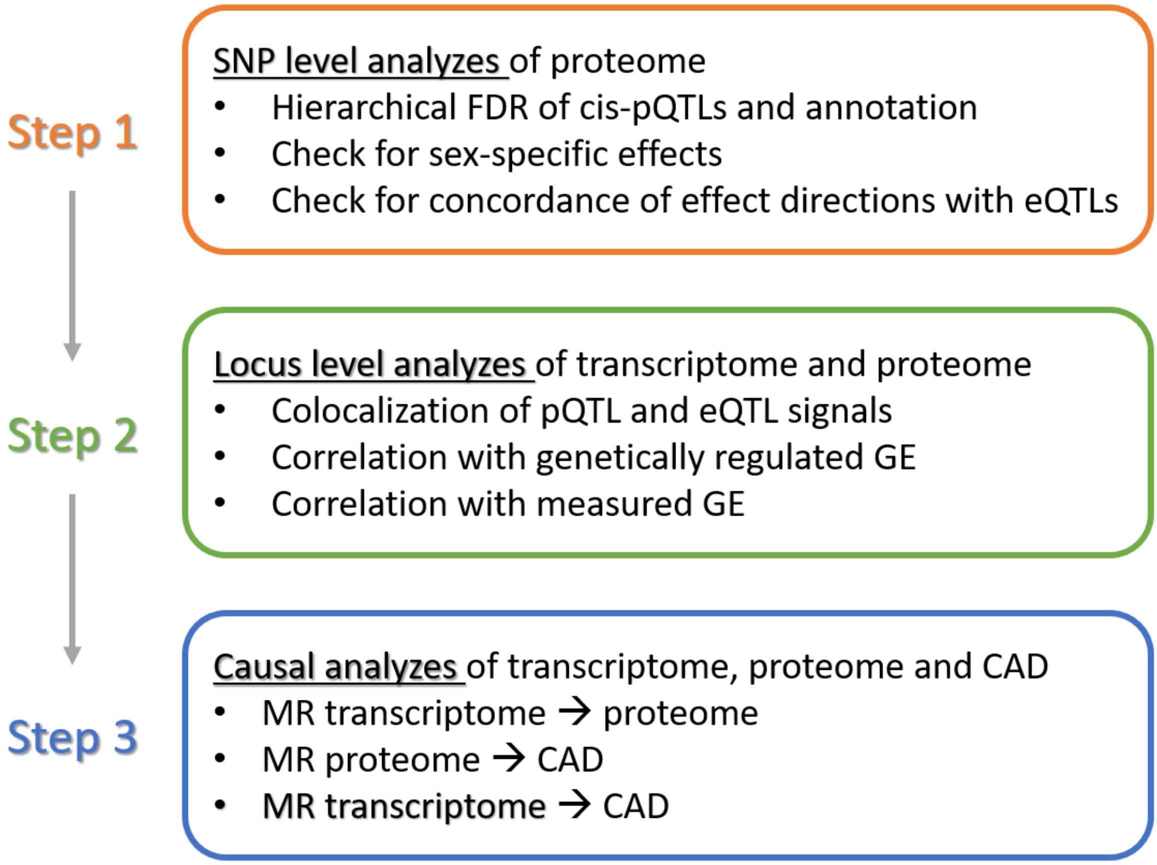

Figure S3: Graphical abstract of the analysis plan. In the first step, we identify associated SNPs per biomarker and compared their association between sexes. We also performed comparisons of eQTL and pQTL effect directions using both GTEx and own GE data. Secondly, we use multiple SNPs at each identified region to (1) analyze the agreement of pQTL and eQTL signals (GTEx, all cis SNPs), and (2) to correlate genetically controlled gene expression (GE) with the protein levels. We validate findings in blood with own raw GE data. Finally, we check if our detected links are causal using Mendelian-Randomization analyses (MR).

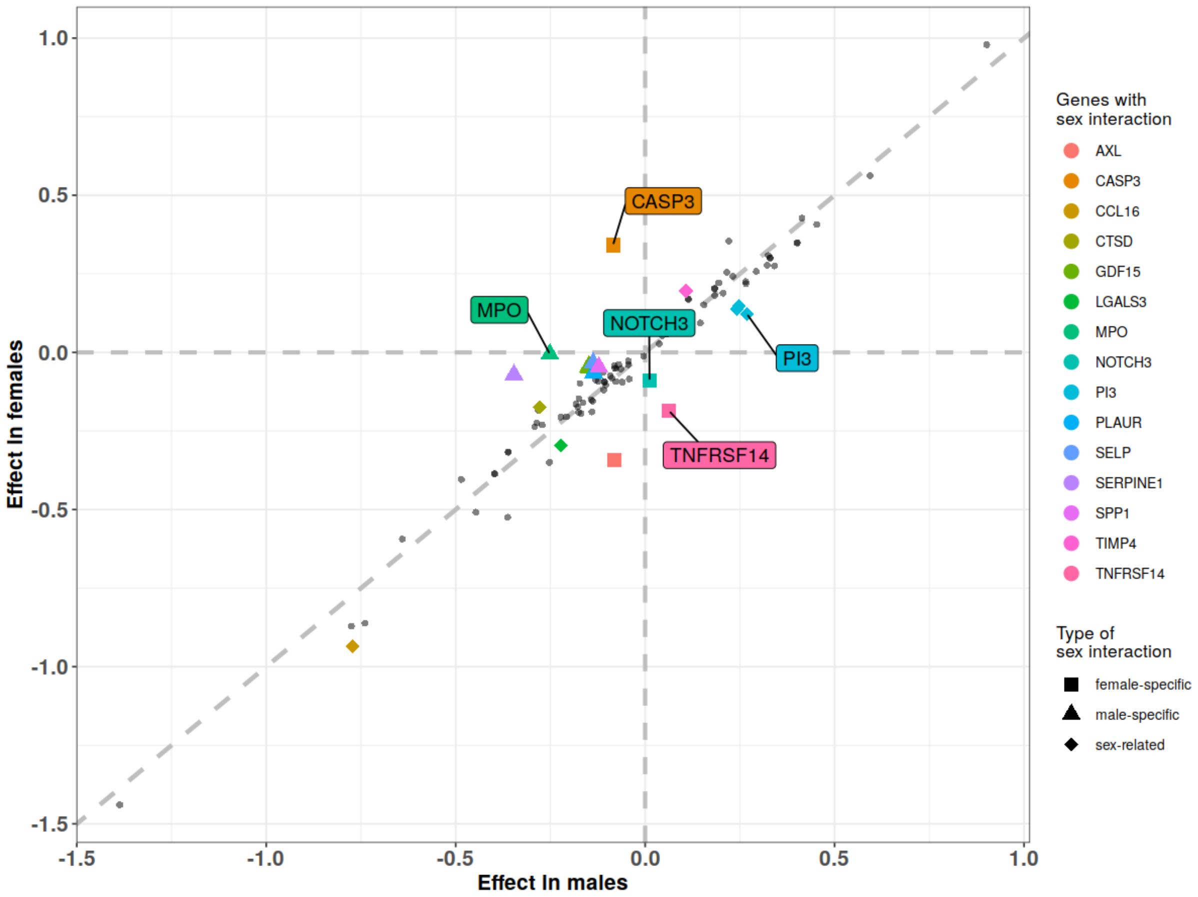

Figure S4: Scatter plot of sex-stratified effect estimates of pQTLs. Proteins with SNP-sex interactions reaching nominal significance are colored, non-significant ones are displayed in black. A gene label was added if the effect difference was significant after FDR correction. Triangles mark male-specific and squares female-specific effects. A diamond indicates sex-related effects, e.g. significant effects in both sexes, but different estimates (stronger effect in one sex).

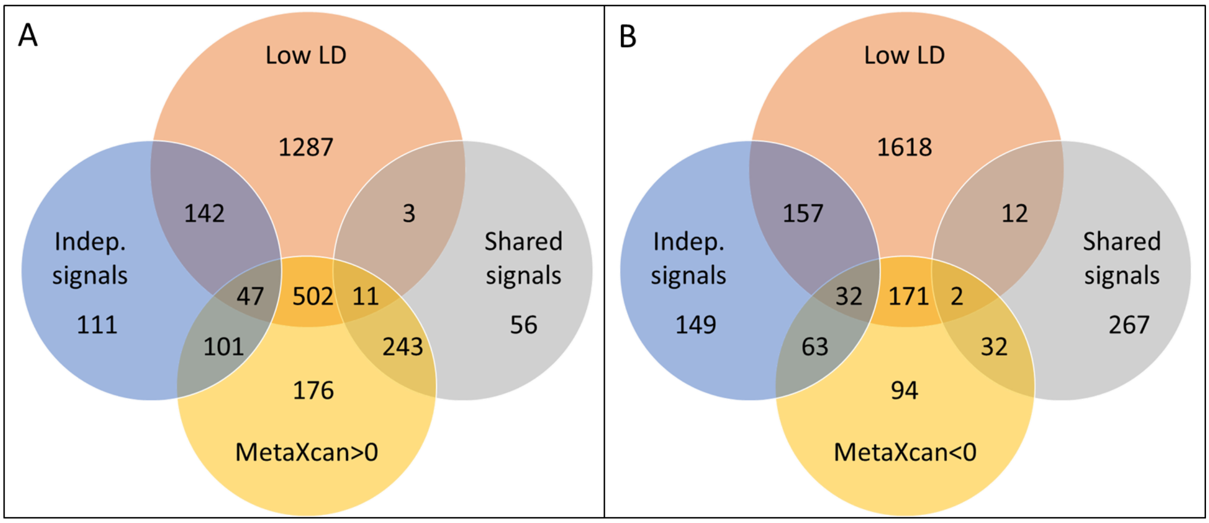

Figure S5: Venn diagram of inter-relationships of pQTLs and eQTLs. We show tissue-protein combinations of possible inter-relationships between pQTLs and eQTLs for different types of analyses. "Low LD" comprises all tissue-protein combinations, whose best-associated eQTL is in low pairwise LD $r^2<0.1$ with the respective pQTL. For colocalization, we report both independent and shared signals (PP3 respectively PP4 $\geq$ 0.75). MetaXcan results were filtered for significant associations after hierarchical FDR. A) Positive association between GE and protein according to MetaXcan. B) Negative association between GE and protein according to MetaXcan.

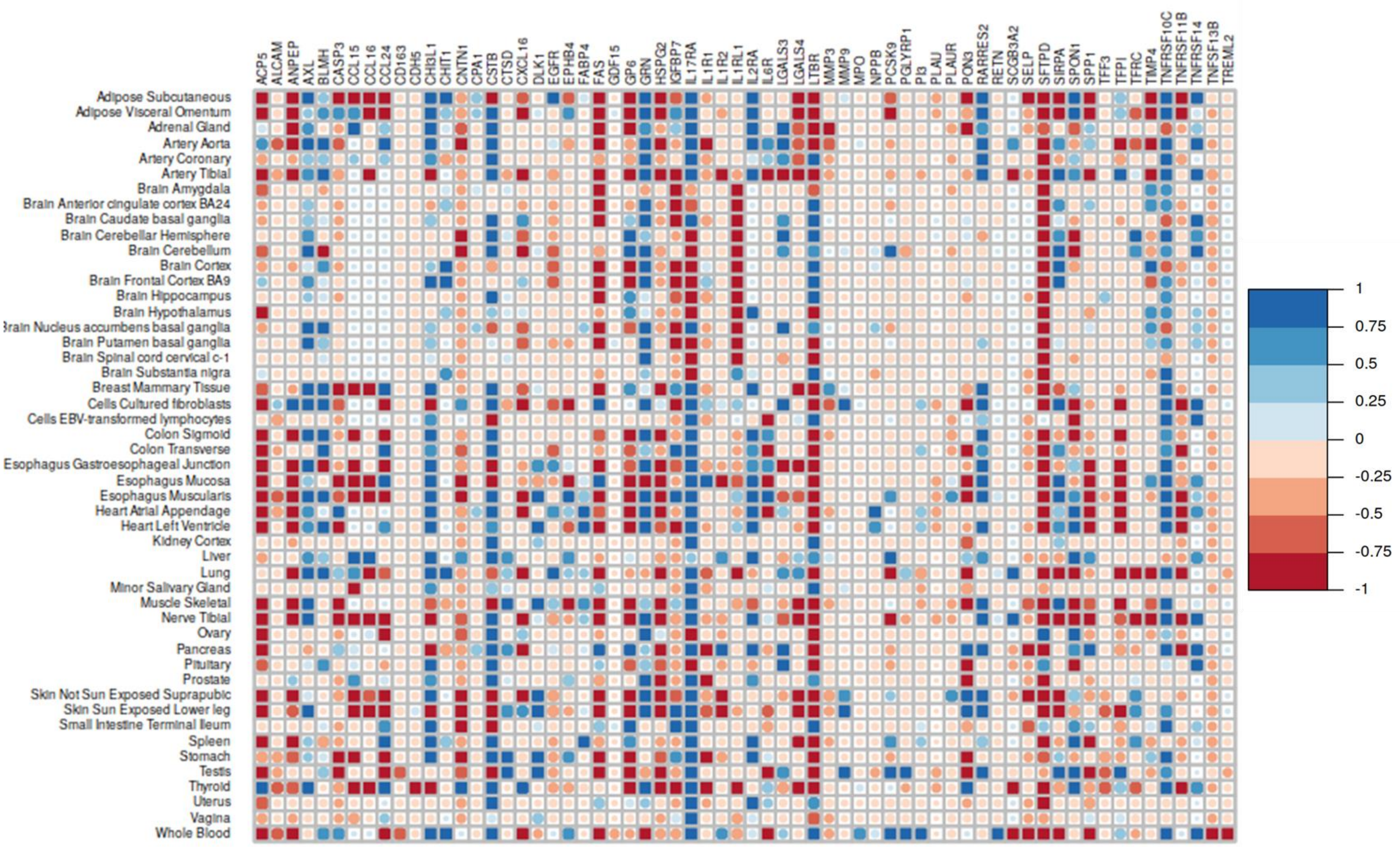

Figure S6: Overview of colocalization results across 48 tissues of GTEx for 63 proteins with pQTLs. Both tissues (rows) and gene names (columns) are ordered alphabetically. Blue indicates colocalization (shared signal), red marks independent signals for gene expression and protein levels. White cells denote combinations without significant eQTLs.

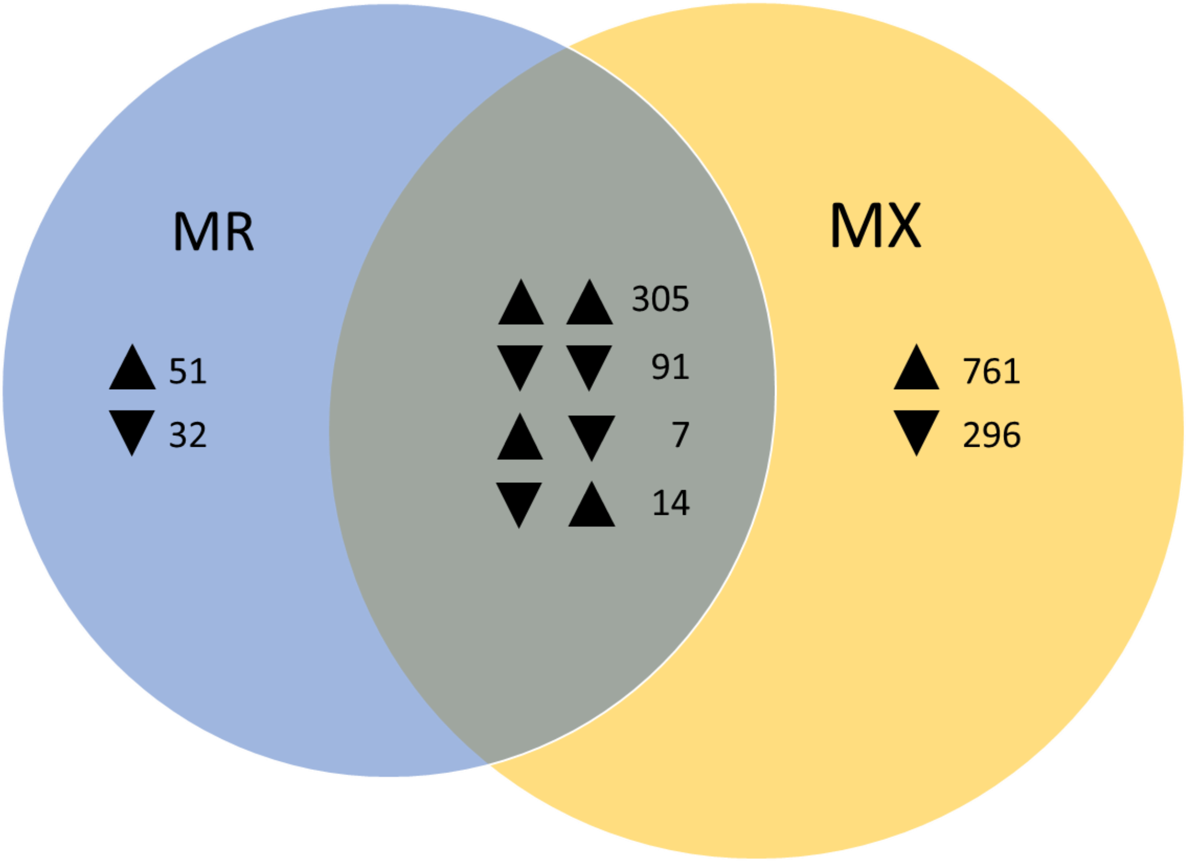

Figure S7: Euler Plot of MetaXcan (MX) association and Mendelian Randomisation (MR) causal estimates. All gene-tissues pairs reaching significance in one of the analyses were displayed (n=1474 for MX, n=501 for MR). The majority of overlapping results have concordant effect direction (n=398). Only for 21 pairs, discordant effects were observed: 14 with positive association, but negative causality, and 7 with negative association and positive causality.

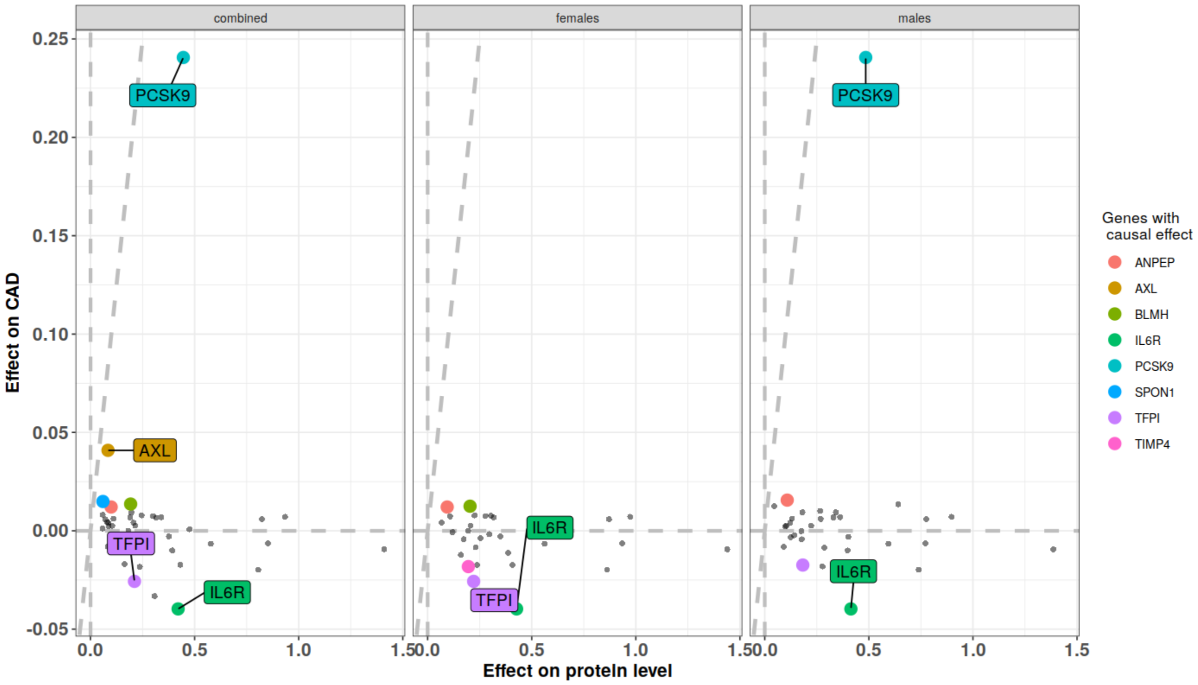

Figure S8: Scatter plot of MR analyses for causal effects of protein levels on CAD in all three settings. Significant causal estimates were found for PCSK9, IL6R, AXL, and TFPI after Bonferroni correction. Four additional proteins showed nominal causal effects ($p<0.05$).
