# Supplementary material for: Genetically regulated gene expression and proteins revealed discordant effects
Source: PLoS One. 2022 May 23;17(5):e0268815. doi: 10.1371/journal.pone.0268815 (PMC9126407; doi:10.1371/journal.pone.0268815)
Supplement: Attachment — Submitted filename: ResponseToReviewers.docx [file pone.0268815.s003.docx]

Point by point response

Pott et al. LWAS Olink biomarker

Reviewer #1:

The authors did an extensive work to study the 92 proteins in the whole blood from 2014 participants in both SNP level and locus level. Of which, 63 proteins were genetically regulated, and 3 sex specific effects were identified. The authors then tested for the colocalization between pQTLs and eQTLs and found evidence of both independent and shared signals. Additionally, the authors tested for the association of genetically regulated gene expression (GE) across tissues and identified 10 proteins of negative correlations. Finally, the authors tested the causal link of genomics, transcriptome, proteome to coronary artery disease (CAD) risk and confirmed a negative causal effect of GE on protein level for 5 genes. The main finding was the gene IL6R.

Comment 1: In the statistical analysis, the population stratification (PS) is not considered. It would be great if the authors can include more details to justify why PS is not a concern in the study. Otherwise, if there exists any PS, not adjusting for it could induce significant bias.

Author reply: The LIFE Adult study is a long-term, population-based cohort study with participants from Leipzig and surrounding areas. In line with this, the genetic homogeneity is high (see new Supplemental Figure 3). We therefore decided to remove the few ethnic outliers and refrained from adjustment for principal components. However, we repeated our association tests for SNPs after hierarchical FDR and adjusted for 10 principle components. The beta estimates were essentially the same, while the standard error increased slightly. All SNPs remained significant according to our hierarchical FDR threshold (see Supplemental Table 4).

Changes in manuscript: We added a PCA plot of our study data as new Supplemental Figure 3 revealing high homogeneity, and included PC adjusted statistics for the lead SNPs in Supplemental Table 4. We also described our approach in the Material section in more detail.

Comment 2: GTEx v8 data has been released for quite a while. I wonder why the authors used v7 instead of v8?

Author reply: We thank the reviewer very much for this suggestion. Previously, we used hg19 SNP data, which is the same build as in GTEx v7. We agree that the most recent version of GTEx should be used and have therefore harmonized our data to hg38 and repeated all our analysis with the harmonized data and GTEx v8.

Changes in manuscript: We updated the methods for the harmonization and GTEx v8 usage. Please note that this has changed all of the tables and figures, while the main message of the paper remains the same.

Comment 3: The proteins in the study were supposed to be cardiovascular (CVD) biomarkers and the authors tried to establish the link from genomics, transcriptome, proteome to CAD risk. I wonder why the authors included CVD-unrelated tissues, especially when testing for the causal path involving CAD, how does the GE and PE in colon, lung and testis contribute to CAD outcome?

Author reply: We agree to discuss this in more detail. Cis-effects have been reported to be shared across tissues, with exception of brain tissues [The GTEx Consortium, DOI: 10.1126/science.aaz1776]. Hence we used also different tissues, partly with larger sample size, which might still detect the true contribution of the gene expression on CAD. Our described links of GE to CAD are mediated by PE, suggesting that increased GE in CAD-unrelated tissues lead to higher blood protein levels, which are then transported to heart tissues, where they increase the risk for a cardiovascular event.

Changes in manuscript: We added a paragraph in the Discussion section.

Comment 4: The authors may need to review and correct the typos and formatting issues throughout. For example, 1) in the abstract, CAD is first mentioned and needs to be defined; 2) The table 2 needs to be re-formatted and the minor allele of CCL15 does not look right. In line 238, the results show ten genes instead of eight; 3) The gene names should be in italics format throughout; 4) The figure 2-4 in the main text are mis-numbered as figure 1-3.

Author reply: We are sorry for these typos.

Changes in manuscript: We checked our manuscript after the extensive changes. We believe we have now eliminated all typos and formatting issues.
