# Supplementary material for: Genetically regulated gene expression and proteins revealed discordant effects
Source: PLoS One. 2022 May 23;17(5):e0268815. doi: 10.1371/journal.pone.0268815 (PMC9126407; doi:10.1371/journal.pone.0268815)
Supplement: Attachment — Submitted filename: ResponseToReviewers.docx [file pone.0268815.s004.docx]

Point by point response

Pott et al. LWAS Olink biomarker – Revision #2

Reviewer #1:

The authors did extensive work to revise the manuscript. Now the manuscript looks great. I do not have further question.

Authors reply: We thank the reviewer very much for the positive evaluation.

Reviewer #2:

In the revised version of their manuscript the authors have addressed the comments raised by the reviewer and doing so the quality and clarity of the manuscript have improved substantially.

I have only a couple of (very) minor further comments:

Authors reply: We thank the reviewer very much for the encouraging evaluation and the helpful comments.

Comment 1: Figure numbers in the main text are still not correct.

Author reply: We are sorry for not checking the created PDF file for this mix-up. For Fig 1, the corresponding text box was accidently deleted. Hence, when converting to a PDF, the auto-numbering used 1 for Fig 2, and so on.

Changes in manuscript: We added the text box for Fig 1 again.

Comment 2: The authors may want to present numbers in a consistent way throughout the manuscript, either as e.g. "2384" or "2,384".

Author reply: We are sorry for this mix-up and present numbers now consistently as “2,014”.

Changes in manuscript: We changed all number so that the thousands separator is consistently a comma.

Comment 3: It seems at several occasions there is a typo with r22 instead of r2 (when referring to LD), and line 313 “data WERE available”.

Author reply: We believe this to be a marked-up problem, as we did not find this typo in the cleaned manuscript. We accidently deleted the superscripted 2, and added it again. However, in the marked-up mode, a superscripted deleted number looks like an underlined number and hence it reads as r^22^ instead of r^2^.

Changes in manuscript: We checked the manuscript for the r^22^ typo and corrected line 313.

Comment 4: The authors may want to check for consistent use of past tense in the material and methods section, e.g. line 135 and line 196 in the revised version of the manuscript.

Author reply: We are sorry for these inconsistencies and corrected them in the manuscript

Changes in manuscript: We checked the Material & Methods section and corrected the grammar if necessary.

Comment 5: Data from the 1000 Genome Project Phase 3 have been used for genotype imputation. I wonder, why this rather old dataset was used instead of a more updated sources for imputation, e.g. the Haplotype reference consortium r1.1 .

Author reply: We were not able to legally update imputation of genetic data of the LIFE consortium to newer references such as HRC or TopMed, as these references are not publicly accessible for imputation on our servers. Therefore, genetic data of the LIFE-consortium would have to be transferred to e.g. Michigan Imputation Server in the United States of America (US) or Sanger Imputation Server in the United Kingdom (UK). For both US and UK, there are still open Data Privacy concerns, as the level of data protection does not comply with the standards required by the European Union (EU). For the US, the Trans-Atlantic Data Privacy Framework is under development, but not yet in place (see https://ec.europa.eu/commission/presscorner/detail/en/ip_22_2087). For the UK, an adequacy decision under the General Data Protection Regulation had been adopted by the EU just last June. However, the final decision for imputation on a server in a country other than an EU member state lies within the LIFE consortia. We will update imputation of the LIFE genetic data once the consortium agrees, or an alternative Imputation Server is available within the EU.

Changes in manuscript: None.

Comment 6: I find the term "best eQTL" a bit unclear and would like the authors to specify whether they refer to strongest/most significant eQTL and/or largest effect size.

Author reply: We meant the cis-eQTL with lowest p-value per gene and tissue.

Changes in manuscript: We added this information in the Material and Method section.

Comment 7: A more cautious wording with regard to Mendelian Randomization may be more adequate, as this methods gives an estimate of causation/causality but does not prove or guarantee such relationship, e.g. line 349 "IL6R was the only protein that showed causality in all three settings".

Author reply: We thank the reviewer for this reminder and agree to tone down the statements on causality.

Changes in manuscript: We used a more cautious wording in the Results section regarding Mendelian Randomization. In addition we added some information why not all settings were tested for this analysis.

Comment 8: In the part of the discussion section where the authors refer to TPM values of several genes in several tissues, many new results are reported (that otherwise have only appeared in supplementary material). I suggest to limit the content of new data presented here and instead report these findings in the results section.

Author reply: In this part of the discussion, we tried to evalute the functional relevance by checking the published expression rate of GTEx v8 of the identified genes by tissues, e.g. do these negative links only occur in tissues with low expressions. Hence, that are not new results. However, we agree to shorten that paragraph by focusing on the main comparisions only.

Changes in manuscript: We shortend the TPM paragraph in the discussion and clarified its reference, GTEx v8.
